# Chromosome-level genome assembly of an endangered plant *Prunus mongolica* using PacBio and Hi-C technologies

**DOI:** 10.1093/dnares/dsad012

**Published:** 2023-05-23

**Authors:** Qiang Zhu, Yali Wang, Ning Yao, Xilu Ni, Cuiping Wang, Meng Wang, Lei Zhang, Wenyu Liang

**Affiliations:** School of Life Sciences, Ningxia University, Yinchuan 750021, China; State Key Laboratory of Efficient Production of Forest Resources, Ningxia Forestry Institute, Yinchuan 750001, China; State Key Laboratory of Efficient Production of Forest Resources, Ningxia Forestry Institute, Yinchuan 750001, China; School of Life Sciences, Ningxia University, Yinchuan 750021, China; School of Ecology and Environment, Ningxia University, Yinchuan 750021, China; College of Biological Science and Engineering, North Minzu University, Yinchuan, 750021, China; School of Life Sciences, Ningxia University, Yinchuan 750021, China; College of Biological Science and Engineering, North Minzu University, Yinchuan, 750021, China; School of Life Sciences, Ningxia University, Yinchuan 750021, China

**Keywords:** *Prunus mongolica*, endangered plant, chromosome-level genome, genome assembly

## Abstract

*Prunus mongolica* is an ecologically and economically important xerophytic tree native to Northwest China. Here, we report a high-quality, chromosome-level *P. mongolica* genome assembly integrating PacBio high-fidelity sequencing and Hi-C technology. The assembled genome was 233.17 Mb in size, with 98.89% assigned to eight pseudochromosomes. The genome had contig and scaffold N50s of 24.33 Mb and 26.54 Mb, respectively, a BUSCO completeness score of 98.76%, and CEGMA indicated that 98.47% of the assembled genome was reliably annotated. The genome contained a total of 88.54 Mb (37.97%) of repetitive sequences and 23,798 protein-coding genes. We found that *P. mongolica* experienced two whole-genome duplications, with the most recent event occurring ~3.57 million years ago. Phylogenetic and chromosome syntenic analyses revealed that *P. mongolica* was closely related to *P. persica* and *P. dulcis*. Furthermore, we identified a number of candidate genes involved in drought tolerance and fatty acid biosynthesis. These candidate genes are likely to prove useful in studies of drought tolerance and fatty acid biosynthesis in *P. mongolica*, and will provide important genetic resources for molecular breeding and improvement experiments in *Prunus* species. This high-quality reference genome will also accelerate the study of the adaptation of xerophytic plants to drought.

## 1. Introduction

Climate change-induced drought is responsible for substantial negative impacts to natural ecosystems, including altering ecosystem structure and function, and decreasing plant productivity, soil fertility, species richness, and plant cover.^1^ Nevertheless, many xeric plants have exhibited adaptability in the face of continued drought due to their unique adaptive traits.^2^ Xeric plants have historically been crucial for the maintenance of ecosystem health and agricultural development in arid regions. However, the distribution and population size of many xeric plant species have declined significantly due to human activities, such as grazing, tourism, and surface mining. This trend is expected to continue as economic and social development continues to reduce and degrade arid wildland habitats. Therefore, it is of critical importance to study and characterize the biological and genetic resources of xeric plants for breeding and other purposes.

The Mongolian almond (*Prunus mongolica*, Rosaceae) is a xeric, diploid (2*n* = 2x = 16)^3^ shrub distributed across the arid regions of western China, including inner and southern Mongolia, Gansu, and Ningxia, among others. *P. mongolica* is extremely drought resistant and grows primarily in arid hilly, mountainous, and desert regions where the annual rainfall ranges between 50 and 200 mm.^4^*P. mongolica* has evolved special genetic resources such as drought resistance, cold resistance, sand resistance, and barren resistance.^5^ Furthermore, *P. mongolica* is highly eco-functional, providing both biomass and medicinal compounds.^6^ The seeds of this species have high food and medicinal value, as the kernels are rich in oil (54.85%) and have been used to treat renal fibrosis.^7^ In China, *P. mongolica* has been listed as an endangered Tertiary relict plant and an endangered second-class protected plant, and has also been listed as ‘vulnerable’ by the International Union for the Conservation of Nature.

A thorough characterization of the *P. mongolica* genome would contribute to our understanding of the phylogeny of Rosaceae; the paleo-floristic, paleo-geographic, and climatic characteristics of the Tertiary Period; and the evolutionary and successional history of the flora endemic to arid central Asia. However, no previous genome-wide investigations of *P. mongolica* have been reported, likely because a high-quality chromosome-level gene map is unavailable. In this study, we assembled a chromosome-level *P. mongolica* genomic assembly using a combination of PacBio high-fidelity (HiFi) reads and Hi-C reads. Using this high-quality genome, we investigated the evolutionary history of *P. mongolica*, and identified a number of candidate genes involved in fatty acid biosynthesis and drought resistance. The genome assembly presented here will provide a valuable resource for the evolutionary study of xerophytic plants as well as for the successful breeding of this ecologically and economically important species.

## 2. Materials and methods

### 2.1. Plant materials and DNA extraction

**Figure 1.**
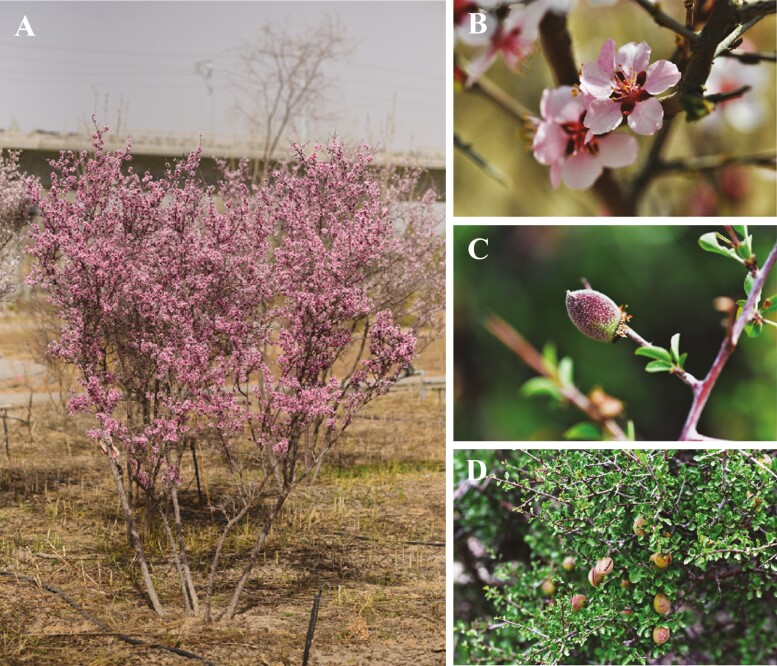
Morphological features of *Prunus mongolica.* (A) Whole plant in flowers, (B) flower, (C) Young fruit, (D) Ripe fruit and branches.

Fresh leaves of mature *P. mongolica* specimens were collected from Yinchuan Botanical Garden (106°10’36’’E,38°25’19’’N), Ningxia Province, northwestern China (Fig. 1). Leaf samples were immediately frozen in liquid nitrogen and stored for further analysis. For whole-genome sequencing, total genomic DNA was extracted from fresh leaves using a modified CTAB method.^8^ After extraction, the concentration, integrity, and purity of the DNA were determined using a NanoDrop spectrophotometer (A260/A280 = 1.8, A260/A230 = 2.0–2.2) and a Qubit.

### 2.2. Genome sequencing

An Illumina genomic library was constructed according to Illumina’s standard protocol. Paired-end (PE) reads (2 × 150 bp) sequenced on an Illumina NovaSeq 6000 platform were used for genomic survey and assessment. To produce CCS reads (HiFi) for contig assembly, the genomes were sequenced using a PacBio Sequel II platform (Pacific Biosciences). For Hi-C sequencing, the chromosomal structure was crosslinked with formaldehyde, and the genomic DNA was digested using HindIII.^9^ After a Hi-C library with a 300–700 bp insert size was constructed, the concentration and insert size were detected using a Qubit2.0 and an Agilent 2100, and the effective concentration was quantified by qPCR. After the libraries were qualified, high-throughput sequencing was performed with an Illumina NovaSeq 6000 platform, with a PE150 reading length.^10^ To aid in gene annotation and phylogenomic analyses, fresh leaves, roots, shoots, flowers, and fruits from the same *P. mongolica* specimen were collected for RNA sequencing (RNA-seq). High-quality RNA-seq libraries were prepared and sequenced with an Illumina NovaSeq 6000 platform. RNA-seq reads were filtered using Trimmomatic^11^ (version 0.36), with default parameters. Low-quality sequencing reads were filtered out and were excluded from further analyses. All sequencing services were provided by Biomarker Technologies Co., Ltd. (Beijing, China).

### 2.3. Genome survey and assembly

Genome size and complexity were estimated based on the k-mer distribution of Illumina short reads. GenomeScope^12^ (version 2.0) was used to count the distribution of 21-mers, with default parameters. A *de novo* assembly of the PacBio HiFi reads was constructed with Hifiasm^13^ (version 0.16), and redundant sequences were filtered out with purge_dups.^14^ CEGMA^15^ (version 2.5) and BUSCO^16^ (version 4) were used to assess the completeness of the genome and gene annotation.

### 2.4. Chromosome assembly using Hi-C

To generate high-quality Hi-C reads for chromosome-level assembly, adapter sequences and low-quality PE reads were removed. The resultant high-quality Hi-C data were truncated at putative Hi-C junctions and mapped to contigs using BWA^17^ (version 0.7.10-r789). HiC-Pro^18^ (version 2.10.0) was used to filter valid reads, and only uniquely mapped PE reads were selected for further analyses. Genome sequences were clustered and ordered onto chromosomes using LACHESIS, with the following parameters: Cluster_min_re_sites = 100, Cluster_max_link_density = 2, order_min_n_res_in_trunk = 15, order_min_n_res_in_shreds = 1.5.

### 2.5. Protein-coding gene prediction

We integrated three approaches to annotate protein-coding genes in the genome: *de novo* prediction, homology search, and transcript-based assembly. The *de novo* gene models were predicted using two *ab initio* gene-prediction software tools, Augustus^19^ (version 3.1.0) and SNAP^20^ (2006-07-28). For the homology-based approach, GeMoMa^21^ (version 1.7) was used to construct a reference gene model using data from several related species (*P. dulcis, P. armeniaca, P. mume*, and *P. persica*). For the transcript-based prediction, RNA-seq data were mapped to the reference genome using Hisat^22^ (version 2.1.0) and assembled by Stringtie^23^ (version 2.1.4). GeneMarkS-T^24^ (version 5.1) was used to predict genes based on the assembled transcripts. PASA^25^ (version 2.4.1) was used to predict genes based on a combination of full-length PacBio transcripts and unigenes assembled by Trinity^26^ (version 2.11). Gene models from these different approaches were combined using EVM^27^ (version 1.1.1) and updated using PASA.

### 2.6. Functional annotation

Functional annotation of *P. mongolica* protein-coding genes was performed by a BLASTP against several public databases, including EggNOG^28^ (5.0, http://eggnog5.embl.de/#/app/home. de/download/eggnog_5.0/), gene ontology^29^ (GO, 20200615, http://geneontology.org), eukaryotic orthologous groups^30^ (KOG, 20110125), Pfam^31^ (version 33.1), TrEMBL,^32^ Non-Redundant^33^ (NR, 202009, ftp://ftp.ncbi.nlm.nih.gov/blast/db), SwissProt^32^ (202005, http://ftp.ebi.ac.uk/pub/databases/swissprot), and Kyoto Encyclopedia of Genes and Genomes^34^ (KEGG, 20191220), with an *E*-value cut-off of 1.0 × 10^−5^.

### 2.7. Identification of repetitive elements

RepeatModeler 2^35^ (version 2.0.1) was used to customize a *de novo* genomic repeat library which could automatically execute two *de novo* repeat-finding programs: RECON^36^ (version 1.0.8) and RepeatScout^37^ (version 1.0.6). To classify the predicted results, RepeatClassifier^35^ was used to search the Dfam^38^ (version 3.5) database. Full-length long terminal repeat retrotransposons (fl-LTR-RTs) were identified using both LTRharvest^39^ (version 1.5.10) (-minlenltr 100 -maxlenltr 40000 -mintsd 4 -maxtsd 6 -motif TGCA -motifmis 1 -similar 85 -vic 10 -seed 20 -seqids yes) and LTR_FINDER^40^ (version 1.07) (-D 40000 -d 100 -L 9000 -l 50 -p 20 -C -M 0.9). The high-quality intact fl-LTR-RTs and the non-redundant LTR library were then produced using LTR_retriever^41^ (version 2.9.0). The sequences flanking both sides of LTRs were extracted and compared using MAFFT^42^ (version 7.205), and the distance was calculated using the Kimura model in EMBOSS^43^ (version 6.6.0). The integration times (million years ago, Mya) of intact LTRs were estimated using the following equation: T = K/2r, where K is the number of nucleotide substitutions per site between each LTR pair, and r is the nucleotide substitution rate (7 × 10^–9^ substitutions per site per year^44^). A non-redundant, species-specific TE library was constructed by combining the *de novo* TE sequence library with data from the Repbase (version 19.06), REXdb (version 3.0) and Dfam (version 3.2) databases. Final *P. mongolica* genomic TE sequences were identified and classified by homology search against the library using RepeatMasker^45^ (version 4.10). Tandem repeats were annotated using Tandem Repeats Finder^46^ (version 409) and the MIcroSAtellite identification tool^47^ (version 2.1).

### 2.8. Identification of pseudogenes and non-coding RNA genes

In this study, we identified several non-coding RNA molecules, including miRNA, rRNA, tRNA, snoRNA, and snRNA. tRNAscan-SE^48^ (version 1.3.1) was used to predict tRNA, using ‘eukaryote’ parameters. Barrnap^49^ (version 0.9) was used to predict rRNA. miRBase^50^ (release 21) was used to predict miRNA. Both snoRNA and snRNA genes were predicted using INFERNAL^51^ (version 1.1) to search the Rfam^52^ (release 14.5) database. GenBlastA^53^ (version 1.0.4) and GeneWise^54^ (version 2.4.1) were used to predict pseudogenes.

### 2.9. Reconstruction of the phylogenetic tree

OrthoFinder^55^ (version 2.5.1) was used to identify homologous genes in *P. mongolica* and 10 other species. The protein sequences of the single-copy orthologs were aligned with MAFFT^43^ (version 7.205), with default parameters. The unique gene families were identified and annotated using the Pfam^56^ (version 33.1) database. GO and KEGG enrichment analyses were carried out using clusterProfiler^57^ (version 3.6.0). To estimate the best substitution models, ModelFinder^58^ was implemented in IQ-TREE^59^ (version 1.6.11). To construct the maximum-likelihood tree, RAxML was used with the best-fit substitution model, with 1,000 bootstrap replicates. Divergence time was estimated using the MCMCTreeR^60^ (version 1.1) program in the Phylogenetic Analysis by Maximum Likelihood software^61^ (PAML, version 4.9i), using three secondary calibration points [*P. mongolica* vs *Amborella trichopoda* (179–199.1), *P. dulcis* vs *P. armeniaca* (3.8–10.2), and *P. dulcis* vs *Fragaria nilgerrensis* (44.5–89.3)] obtained from the TimeTree database (http://www.timetree.org/). The graphical phylogenetic tree was constructed using MCMCTreeR.

### 2.10. Analyses of gene-family expansion and contraction

Based on the identified gene families, the phylogenetic tree, and the predicted divergence times, we used Computational Analysis of gene Family Evolution^62^ (CAFE, version 4.2) to analyse gene-family expansion and contraction. In CAFE, a random birth and death model is used to study gene gain or loss in gene families across a specified phylogenetic tree. Then, a conditional *P*-value is calculated for each gene family, and families with conditional *P*-values < 0.05 are considered to have an accelerated rate of gene gain or loss. The expanded and contracted gene families in *P. mongolica* (*P*-value ≤ 0.05) were subjected to KEGG pathway enrichment analysis. This method utilized hypergeometric test algorithms, and the *Q*-value (False Discovery Rate, FDR) was calculated to adjust the *P*-value using the R package *q*-value (https://github.com/StoreyLab/qvalue).

### 2.11. Positively selected genes

Based on the phylogenetic tree, we selected five closely related species (*P. armeniaca, P. mume, P. dulcis, P. mongolica*, and *P. persica*) to estimate the ratio (ω) of non-synonymous (Ka) to synonymous (Ks) nucleotide substitutions using PAML^63^ (version 4.9e). ClusterProfiler^57^ (version 3.6.0) was used to perform GO and KEGG enrichment analyses on the positively selected genes of *P. mongolica*.

### 2.12. Synteny analysis and whole-genome duplication

Synteny analysis was performed using Diamond^64^ (version 0.9.29.130). MCScanX^65^ was used to identify collinear blocks. The synonymous mutation rate (Ks) and fourfold synonymous third-codon transversion rate (4DTv) are commonly used to identify whole-genome duplications (WGDs). Here, wgd software^66^ (version 1.1.1) was used with a custom script (https://github.com/JinfengChen/Scripts) to identify WGD events in *P. mongolica*.

### 2.13. Identification of fatty acid biosynthesis and drought resistance genes

Drought resistance, fatty acid biosynthesis, and triglyceride biosynthesis genes in the *P. mongolica* genome were identified by homologous search against the *Arabidopsis thaliana* genome. The candidate genes were further filtered by checking their Enzyme Commission number.

## 3. Results and discussion

### 3.1. High-quality *P. mongolica* genome assembly

Prior to assembly, a total of 18.18 Gb of Illumina data were generated (Supplementary Table S1). In total, 1,574,319,910 k-mers (k = 21) were identified, and a major peak was observed at a k-depth of 33 (Supplementary Fig. S1). After removing low-frequency k-mers, the *P. mongolica* genome size was estimated to be 226.47 Mb, with a high level of heterozygosity (1.61%) and a large percentage of repeat sequences (39.01%). To assemble this highly complex genome, we obtained a total of 9.59 Gb (×42.34 genome coverage) of HiFi long reads, with a maximum read length of 42,695 bp and an average read length of 15,176 bp (Supplementary Tables S1 and S2). Using Hifiasm stitching, the total length of the genomic contig sequence was found to be 271.26 Mb, with a contig N50 of 24.33 Mb. After removal of haplotigs and overlaps, the total length of the genomic contig sequence was 233.17 Mb, with a contig N50 of 24.33 Mb (Supplementary Table S2). To validate the quality of the Hifiasm stitching, we first mapped the Illumina reads back to the assembly and obtained an overall mapping rate of 98.36%, 10-fold minimum genome coverage of 97.54%, and an average sequencing depth of ×73 (Supplementary Table S3). Furthermore, we obtained a BUSCO completeness score of 98.76% and CEGMA indicated that 98.47% of the assembled *P. mongolica* genome was reliably annotated (Supplementary Tables S4 and S5). Taken together, these results suggest that our *P. mongolica* genome assembly was high quality and complete.

**Figure 2.**
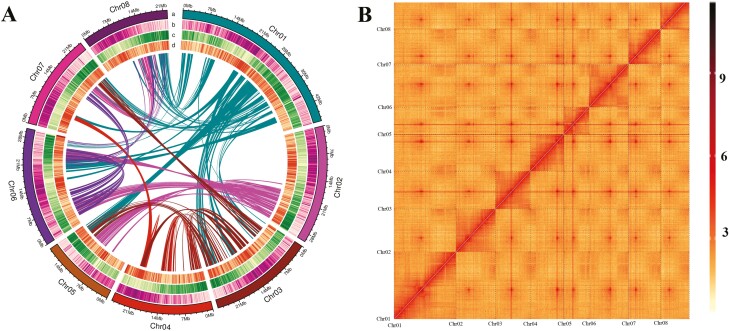
Overview of *Prunus mongolica* and its genome. (A) Landscape of the *P. mongolica* genome. The tracks from outer to inner circles indicate the following: a, chromosome ideograms; b, TE (transposable elements) density; c, gene density; d, GC content. (B) Heatmap showing Hi-C interactions at a resolution of 300 kb.

To reconstruct a chromosome-level assembly, we generated 31.49 Gb (×139.04 genome coverage) of Hi-C clean reads (Supplementary Table S1) and anchored the contigs onto pseudochromosomes using the Hi-C scaffolding approach. Ultimately, 227,864,786 bp of sequences (97.73% of the entire assembly) were assigned to eight pseudochromosomes (Fig. 2A), corresponding to the haploid chromosome number of *P. mongolica*. The lengths of the pseudochromosomes ranged from 18,973,745 to 47,189,407 bp (Supplementary Table S6). The corresponding heat map revealed that the eight chromosomal groupings were clearly distinguishable and that all pseudochromosomes exhibited a well-organized diagonal pattern of intra-chromosomal interactions (Fig. 2B and Supplementary Fig. S2), suggesting a high-quality Hi-C-assisted genome assembly. The length of sequences whose order and orientation could be determined was 225,330,101 bp, accounting for 98.89% of the total length of mapped chromosome sequences. The final genome assembly contained very few gaps, with an average of seven gaps per pseudochromosome. The contig and scaffold N50 values were 24,328,480 bp and 26,540,977 bp, respectively (Table 1 and Supplemental Table S7). Taken together, these statistics verified that our genome assembly was precise, complete, and of high quality at the chromosome scale.

**Table 1.** Global statistics of *Prunus mongolica* genome assembly and annotation

Parameter	Size or number
Estimate of genome size (survey), Mb	226,470,058
Assembled genome size, bp	233,169,053
Total length of contigs, bp	233,168,353
Total number of contigs	91
N50 of contigs, bp	24,328,480
Largest contig, bp	30,055,353
Total length of scaffolds, bp	233,169,053
Total number of scaffolds	84
N50 of scaffolds, bp	26,540,977
Largest scaffold, bp	47,189,607
GC content, %	38.20
Complete CEGMA, %	98.47
Complete BUSCOs, %	98.76
Total length of repeat, bp	88,539,904
Repeat density, %	37.97
Long terminal repeat (LTR) density, %	17.92
Microsatellite repeat density, %	0.94
Number of protein-coding genes	23,798
Number of annotated genes	23,702
Number of rRNA	832
Number of tRNA	625
Number of miRNAs	217
Number of snRNAs	283
Number of snoRNAs	491
Number of pseudogenes	161

### 3.2. Annotation of the *P. mongolica* genome

We annotated a total of 88,539,904 bp of repetitive sequences, accounting for 37.96% of the entire genome (Supplementary Table S8). Transposable elements (TEs) were the most abundant class of repeats, spanning 59,900,478 bp or 25.68% of the genome. Long terminal repeat-retrotransposons (LTR-RTs) were the next most abundant class of repeats (27,352,348 bp or 11.72% of the genome), including Ty1-Copia superfamily retrotransposons (4.84%), Ty3-Gypsy superfamily retrotransposons (6.53%), and other LTR elements (0.36%). A total of 166,321 tandem repeats were also identified, accounting for 14,174,342 bp or 6.08% of the genome. In addition, we identified 28,806,220 bp (12.35% of the genome) of unknown repetitive sequences, which may be specific to *P. mongolica*.

By combining transcriptome-based, homology-based, and *ab initio* methods, a total of 23,798 protein-coding genes were predicted, of which 23,573 (99.05%) were located on the eight pseudochromosomes (Table 1, Supplementary Table S9, and Supplementary Fig. S3A). The average gene length was 3,448.46 bp with 5.67 exons per gene, with an average exon length of 1,793.77 bp (Supplementary Table S10 and Supplementary Fig. S3B–D). Besides the protein-coding genes, we also identified 2,448 small RNAs and 161 pseudogenes. Of the small RNAs, we identified 625 tRNAs, 832 rRNAs, 217 microRNAs, 283 snRNAs, and 491 snoRNAs (Table 1). A BUSCO completeness score of 98.88% was obtained at the gene model level, indicating the high completeness of gene annotation (Supplementary Table S4). Taken together, these statistics indicate the high accuracy of gene prediction across the *P. mongolica* genome.

Searching these genes against the Pfam, Swiss-Prot, TrEMBL, EggNOG (Supplementary Fig. S4A), and NR (Supplementary Table S11) databases resulted in the functional annotation of 88.62%, 80.99%, 99.58%, 84.71%, and 99.07% genes, respectively. We further annotated these genes using the KOG, GO (Supplementary Fig. S4B), and KEGG databases (Supplementary Table S11).

### 3.3. Evolutionary history of *P. mongolica*

**Figure 3.**
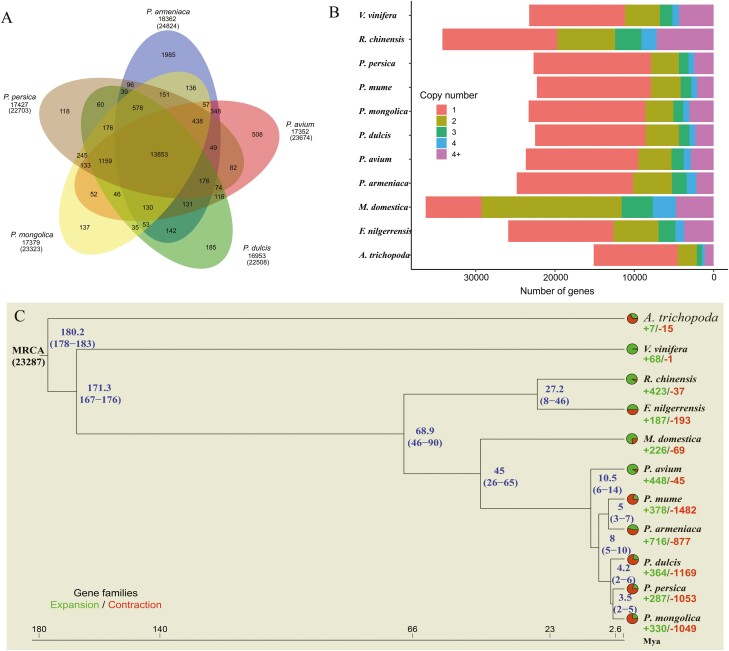
(A) Venn diagram of gene families cluster in five prunus species. (B and C) phylogenetic analysis, divergence time estimates, and the number of gene copies and their distribution among 11 plant species. The tree was constructed on the basis of 1,538 single-copy truly orthologous genes. Divergence times (Mya) are indicated by the blue numbers beside the branch nodes. The numbers of gene-family expansion and contraction events are indicated by green and red numbers, respectively, on each species branch. Pie charts show the proportions of gene families that underwent expansion or contraction. MRCA, most recent common ancestor.

To investigate the evolutionary history of *P. mongolica*, we compared the *P. mongolica* genome to eight closely related Rosaceae plants (*P. persica*, *P. avium*, *P. mume*, *P. dulcis*, *P. armeniaca*, *Malus domestica*, *Rosa chinensis*, and *F. nilgerrensis*) and two outgroups (*A. trichopoda* and *Vitis vinifera*) (Supplemental Table S12). Among these 11 plant species, 33,254 orthologous families and 1,552 single-copy families were identified. Based on the single-copy genes, a maximum likelihood-based phylogenetic analysis was conducted, revealing that the most recent common ancestor of the 11 species contained 23,287 gene families and 1,538 high-quality single-copy orthologous genes (Fig. 3C). The results further indicated that *P. mongolica* is most closely related to *P. persica* and *P. dulcis*. Molecular dating using *A. trichopoda* and *V. vinifera* for fossil calibration indicated that *P. mongolica*-*P. dulcis* emerged 2–6 Mya and that *P. mongolica*-*P. persica* emerged 2–5 Mya.

Among the 11 species, all protein-coding genes were clustered into 28,283 orthogroups based on sequence homology. A total of 8,272 gene families were shared by all 11 species, and 25 *P. mongolica-*specific gene families were found (Supplementary Fig. S5 and Supplementary Table S13). Comparison of gene families among the five *Prunu*s species revealed that 13,853 gene families were shared among *P. mongolica*, *P. persica*, *P. avium*, *P. mume*, *P. dulcis*, and *P. armeniaca*, while 137 gene families were specific to *P. mongolica* (Fig. 3A). We also identified 330 expanded gene families and 1,094 contracted gene families (Fig. 3B and C, Supplementary Figs. S6 and S7), suggesting that the majority of gene families in *P. mongolica* contracted, rather than expanded, during adaptive evolution.

GO enrichment analysis revealed that the *P. mongolica*-specific gene families were primarily enriched in the biological functions of metabolic process, cellular process localization, membrane part, cell part, binding, and catalytic activity, among others (Table 2). KEGG enrichment analysis revealed that these species-specific gene families were primarily enriched in the metabolic pathways of pyrimidine metabolism, protein processing in endoplasmic reticulum, butanoate metabolism, propanoate metabolism, terpenoid backbone biosynthesis, lysine degradation, fatty acid degradation, valine, leucine and isoleucine degradation, fatty acid metabolism, and pyruvate metabolism, among others (Table 3). KEGG enrichment analysis of expanded gene families found that these were enriched in the cyanoamino acid, tryptophan, arginine, proline, phenylalanine, stilbenoid, and starch and sucrose metabolism pathways (Supplementary Fig. S6E). The majority of the contracted gene families were enriched in sesquiterpene and triterpene biosynthesis, homologous recombinant, endocytosis, and starch and sucrose metabolism (Supplementary Fig. S7E). We also identified 350 positively selected genes in *P. mongolica* (Supplementary Table S14) and subjected these to GO and KEGG enrichment analyses (Supplementary Fig. S8). KEGG enrichment analysis indicated that the majority of positively selected genes were enriched in starch and sucrose metabolism, RNA degradation, sphingolipid metabolism, glycerolipid metabolism, and pyrimidine metabolism. These metabolic processes may be related to the characteristic cold and drought tolerance exhibited by *P. mongolica*.

**Table 2.** GO enrichment analysis of *Prunus mongolica*-specific gene

GO_classify 1	GO_classify 2	All gene	Specific
Cellular component	Extracellular region	501	0
Cellular component	Nucleoid	18	0
Cellular component	Cell junction	175	0
Cellular component	Membrane-enclosed lumen	450	0
Cellular component	Protein-containing complex	2,064	0
Cellular component	Organelle	6,376	0
Cellular component	Other organism	141	0
Cellular component	Other organism part	141	0
Cellular component	Extracellular region part	69	0
Cellular component	Organelle part	2,418	0
Cellular component	Synapse part	2	0
Cellular component	Synapse	3	0
Cellular component	Symplast	42	0
Cellular component	Supramolecular complex	125	0
Molecular function	Structural molecule activity	375	0
Molecular function	Transporter activity	1,039	0
Molecular function	Antioxidant activity	127	0
Molecular function	Protein tag	13	0
Molecular function	Cargo receptor activity	2	0
Molecular function	Translation regulator activity	6	0
Molecular function	Nutrient reservoir activity	61	0
Molecular function	Molecular transducer activity	179	0
Molecular function	Toxin activity	3	0
Molecular function	Molecular function regulator	333	0
Molecular function	Molecular carrier activity	24	0
Molecular function	Transcription regulator activity	724	0
Biological process	Reproduction	441	0
Biological process	Immune system process	69	0
Biological process	Behaviour	5	0
Biological process	Cell proliferation	9	0
Biological process	Carbon utilization	7	0
Biological process	Nitrogen utilization	8	0
Biological process	Reproductive process	438	0
Biological process	Biological adhesion	2	0
Biological process	Signalling	535	0
Biological process	Multicellular organismal process	488	0
Biological process	Developmental process	680	0
Biological process	Growth	79	0
Biological process	Locomotion	14	0
Biological process	Pigmentation	1	0
Biological process	Rhythmic process	22	0
Biological process	Response to stimulus	2,092	0
Biological process	Multi-organism process	279	0
Biological process	Biological regulation	3,177	0
Biological process	Cellular component organization or biogenesis	1,586	0
Biological process	Detoxification	150	0
Biological process	Localization	1,841	2
Cellular component	Cell	8,596	4
Cellular component	Cell part	8,596	4
Cellular component	Membrane	6,095	7
Cellular component	Membrane part	5,331	7
Biological process	Cellular process	8,531	19
Molecular function	Catalytic activity	8,741	29
Biological process	Metabolic process	8,538	29
Molecular function	Binding	10,468	30
**#Total_gene**		**19,944**	**131**

**Table 3.** KEGG enrichment analysis of *Prunus mongolica*-specific gene

ID	Description	GeneRatio	BgRatio	enrich_factor	pvalue	qvalue	geneID
ko00240	Pyrimidine metabolism	4/51	114/9379	6.45	0.0033	0.0456	Pmo02G005780 Pmo02G008730 Pmo04G020390 Pmo07G001640
ko00650	Butanoate metabolism	2/51	33/9379	11.15	0.0137	0.0941	Pmoptg000086l_1G000010 Pmoptg000095l_1G000010
ko00640	Propanoate metabolism	2/51	49/9379	7.51	0.0289	0.1014	Pmoptg000086l_1G000010 Pmoptg000095l_1G000010
ko00900	Terpenoid backbone biosynthesis	2/51	53/9379	6.94	0.0335	0.1014	Pmoptg000086l_1G000010 Pmoptg000095l_1G000010
ko00310	Lysine degradation	2/51	56/9379	6.57	0.0370	0.1014	Pmoptg000086l_1G000010 Pmoptg000095l_1G000010
ko00071	Fatty acid degradation	2/51	68/9379	5.41	0.0526	0.1120	Pmoptg000086l_1G000010 Pmoptg000095l_1G000010
ko00280	Valine, leucine and isoleucine degradation	2/51	75/9379	4.9	0.0626	0.1223	Pmoptg000086l_1G000010 Pmoptg000095l_1G000010
ko01212	Fatty acid metabolism	2/51	86/9379	4.28	0.0794	0.1358	Pmoptg000086l_1G000010 Pmoptg000095l_1G000010
ko00620	Pyruvate metabolism	2/51	108/9379	3.41	0.1166	0.1772	Pmoptg000086l_1G000010 Pmoptg000095l_1G000010
ko04141	Protein processing in endoplasmic reticulum	4/51	381/9379	1.93	0.1519	0.1814	Pmo03G005840 Pmo03G005850 Pmo03G005860 Pmo07G002920
ko00380	Tryptophan metabolism	2/51	128/9379	2.87	0.1534	0.1814	Pmoptg000086l_1G000010 Pmoptg000095l_1G000010
ko00630	Glyoxylate and dicarboxylate metabolism	2/51	131/9379	2.81	0.1591	0.1814	Pmoptg000086l_1G000010 Pmoptg000095l_1G000010
ko00460	Cyanoamino acid metabolism	2/51	179/9379	2.05	0.2543	0.2677	Pmo06G015160 Pmo08G002680
ko01200	Carbon metabolism	2/51	310/9379	1.19	0.5065	0.4950	Pmoptg000086l_1G000010 Pmoptg000095l_1G000010
ko00940	Phenylpropanoid biosynthesis	2/51	362/9379	1.02	0.5913	0.5395	Pmo06G015160 Pmo08G002680
ko00500	Starch and sucrose metabolism	2/51	453/9379	0.81	0.7135	0.6102	Pmo06G015160 Pmo08G002680
ko04144	Endocytosis	1/51	276/9379	0.67	0.7829	0.6302	Pmo07G002920
ko03040	Spliceosome	1/51	350/9379	0.53	0.8570	0.6515	Pmo07G002920

*P. mongolica* grows in harsh, arid environments characterized by drought, cold temperatures, high winds, and sandblasting, among other stressors. Such harsh environmental conditions can negatively impact normal physiological functions, metabolic dynamics, and cell membrane structure and permeability, resulting in damage to protoplasts and deranged signalling pathways.^67,68^ Over long-term evolution, the gene families specific to *P. mongolica* likely evolved to enhance stress resistance by regulating metabolic pathways such as pyrimidine metabolism, protein processing in endoplasmic reticulum, and butanoate metabolism, among others. Additionally, in response to drought and cold stress, *P. mongolica* experienced expansion in gene families related to osmoregulation, scavenging radicals, and protecting cell structures through proline and sucrose metabolism. According to GO analysis, the positively selected genes in *P. mongolica* were found to be enriched in the biological functions of biological process, cellular component, and molecular function. Similarly to the species-specific and expanded gene families, KEGG pathway analysis of positively selected genes revealed that these genes were enriched in the same metabolic pathways, including starch and sucrose metabolism, pyrimidine metabolism, RNA degradation, sphingolipid metabolism, and glycerolipid metabolism, among others. Both positive transcriptional regulation and membrane stabilization are critical responses to environmental stress.^69–71^ It appears that *P. mongolica* maintains normal physiological functioning under harsh climatic conditions through the expression and regulation of a variety of species-specific, expanded, and positively selected genes. Furthermore, the expansion and contraction of gene families and positively selected genes may have contributed to the phenotypic diversification and speciation of *P. mongolica*.

**Figure 4.**
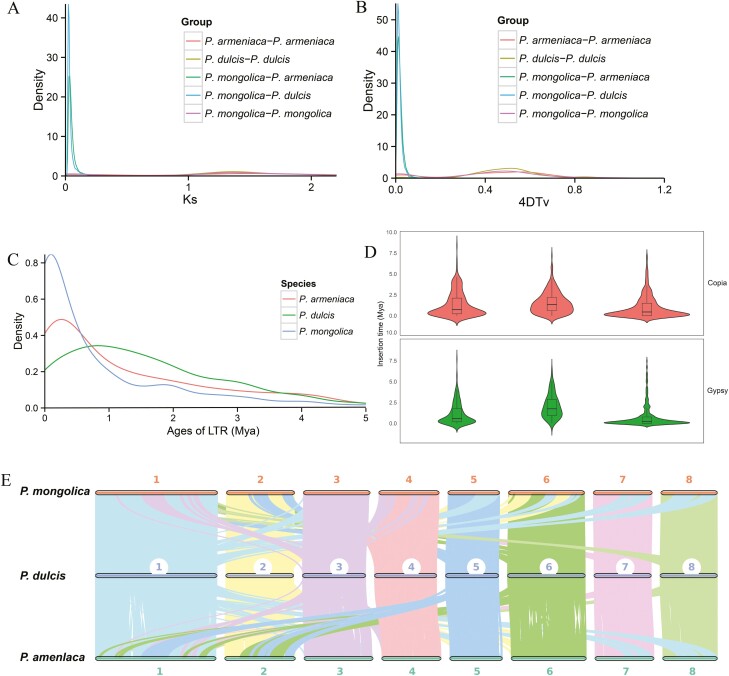
(A) Distribution of the synonymous substitution rate (Ks) between *P. mongolica* and *P. dulcis*, *P. armeniaca*. (B) Genome duplication in *P. mongolica* and genomes of related species as revealed by 4DTv analyses. (C) Distribution of insertion ages of LTR retrotransposons in the genomes of *P. mongolica* and genomes of related species. LTR, long terminal repeat; Mya, million years ago. (D) Copia and Gypsy elements insertion time. (E) MCscanX identified synteny blocks between *P. mongolica*, *P. dulcis*, and *P. armeniaca*.

WGD events (polyploidization) have played a major role in the evolutionary history of angiosperms.^72,73^ In order to study the evolutionary relationships between *P. mongolica* and other plant species, we measured the synonymous substitution rates (Ks) of orthologous gene pairs. On the basis of the distribution of Ks values of ~0.05 and ~1.37 between orthologs, two WGD events were identified in the *P. mongolica* genome, corresponding to divergences at ~3.57 and ~97.86 Mya, respectively. Furthermore, the analysis revealed peaks of ~0.025 for *P. mongolica*-*P. dulcis* and ~0.032 for *P. mongolica*-*P. armeniaca*, corresponding to divergences at ~1.79 and ~2.28 Mya, respectively (Fig. 4A). Analysis of the distribution of sequence divergence values for syntenic duplicate genes revealed two significant peaks for the *P. mongolica* genome (4DTv ~0.018 and ~0.488; Fig. 4B), further confirming that *P. mongolica* had experienced two WGDs. A divergence peak value (4DTv ~0.01 and ~0.002) was observed for *P. dulcis* and *P. armeniaca* (Fig. 4B), suggesting that *P. mongolica* diverged from *P. armeniaca* later than from *P. dulcis.*

LTR retrotransposons are the most abundant group of TEs in plants, and are considered the ‘engine’ of plant evolution.^74^ We found that the majority of LTR-RT insertion events in the *P. mongolica* genome occurred <1 Mya. Furthermore, the genomes of *P. armeniaca*, *P. dulcis*, and *P. mongolica* carried younger LTR-RTs, and the greatest proportion of LTR-RTs exhibited insertion times of ~0.25, 0.83, and 0.1 Mya, respectively (Fig. 4C), with Copia and Gypsy element insertion times of 0.98 and 0.75 Mya (Fig. 4D). This phenomenon may have resulted from rapid environmental change, such as desertification and drought, with the recent burst of LTR-RT insertions and gene duplications coinciding with the aridification of inland Asia during the late Cenozoic.^75^ These results suggest that the substantial increase LTR-RT insertions and tandem gene duplications within the *P. mongolica* genome may have contributed to the expansion of its genome and its adaptation to arid environments.

Synteny block analysis is often conducted to assess assembly quality and to investigate the evolutionary history of related species.^76^ To study colinear relationships within the *P. mongolica*, *P. armeniaca*, and *P. dulcis* genomes, we identified similar gene pairs using Diamond and syntenic blocks using MCScanX (Fig. 4E). On the basis of the order of orthologous genes, a total of 380 and 256 syntenic blocks were identified between *P. mongolica*-*P. armeniaca* and *P. mongolica*-*P. dulcis*, corresponding to 33,908 and 34,680 gene pairs in *P. mongolica*-*P. armeniaca* and *P. mongolica*-*P. dulcis*, respectively. The frequency of large-scale fragment rearrangements was determined in the *P. mongolica*, *P. armeniaca*, and *P. dulcis* genomes, including inversions and translocations, indicating that *P. mongolica*-*P. dulcis* had higher collinearity than *P. mongolica*-*P. armeniaca*, consistent with their close phylogenetic relationship as members of the *Prunus* clade (Supplementary Fig. S9).

### 3.4. Drought resistance and fatty acid biosynthesis genes

With an ever-increasing amount of publicly available data and published research, the model plant *Arabidopsis* (*A. thaliana*) continues to offer a convenient mechanism to identify and characterize functional genes and molecular mechanisms in other eukaryotic organisms.^77^ For example, in *Rubus corchorifolius*, key genes involved in anthocyanin and lignin biosynthesis were identified through comparison with *Arabidopsis* genes.^78^ In *Euphorbia lathyris*, genes related to lipid metabolism were identified by performing homology searches against the *Arabidopsis* genes involved in the biosynthesis of fatty acids and triacylglycerols.^79^

Here, we performed a homology search against the *Arabidopsis* genome and identified a total of 19 and 29 genes likely related to drought resistance and fatty acid/triglyceride biosynthesis, respectively, across the *P. mongolica* genome (Table 4 and Supplementary Table SWGD15). Among these drought resistance genes, *AVP1 (Arabidopsis vacuolar H+-pyrophosphatase gene), CDKC2 (Cyclin dependent kinase group C2)* and *Sucrose synthase 3* (*SUS3)* had two copies; Alcohol dehydrogenase 1 (*ADH1)* had three copies; and Early-responsive to dehydration *(ERD9)* had six copies. All other genes had only one copy. *AVP1*, vacuolar proton-pumping pyrophosphatase (H^+^ -PPase) gene, has been shown to increase plant growth under both stressed and unstressed conditions in *Arabidopsis*.^80^*CDKC2*, a cyclin-dependent protein kinase, enhances plant stress tolerance by regulating the phosphorylation of SR (Serine/arginine-rich)-splicing factors.^81^ In *Arabidopsis*, this phosphorylation triggers the alternative splicing of pre-mRNAs and of stress-related genes, resulting to the induction of the stress response.^81^*SUS3*, one of the key enzymes in sucrose metabolism, is highly responsive to both internal and external environmental signals and can dramatically alter development and stress acclimation.^82^*ADH1*, of which there are three copies in *P. mongolica*, has been found to improve resistance to both biotic and abiotic stressors when overexpressed in *Arabidopsis*.^83^*ERD9*, of which there are six copies in *P. mongolica*, is the core environmental stress response gene in plants.^84^ Among these fatty acid biosynthesis genes, only *Acetyl CO-enzyme A carboxylase subunit* (*CAC2)* had two copies and the other genes had only one copy. These candidate genes are likely to prove useful for the future study of drought tolerance and fatty acid biosynthesis in *P. mongolica*, and will provide important genetic resources for molecular breeding experiments.

**Table 4.** Genes involved in drought resistance in *Prunus mongolica* genome

Genes	Full name	EC number	Candidate genes
ADH1	Alcohol dehydrogenase 1	EC:1.1.1.1	Pmo08G001370, Pmo08G001390, Pmo08G001410
AO4	Aldehyde oxidase 4	EC:1.2.1.28	Pmo06G013050
AVP1	(AVP1)	EC:7.1.3.1	Pmo03G007910, Pmo07G022760
β-AM1	β-Amylase 1	EC:3.2.1.2	Pmo01G004640
CDKC2	Cyclin dependent kinase group C2	EC:2.7.11.22	Pmo01G015660, Pmo06G016710
CER1	Eceriferum 1	EC:4.1.99.5	Pmo02G009550
CHIA	Chitinase A	EC:3.2.1.14	Pmo02G026230
ERD9	Early-responsive to dehydration	EC:2.5.1.18	Pmo04G013040, Pmo04G013050, Pmo04G013060, Pmo04G013070, Pmo04G013080, Pmo04G013090
GolS2	Galactinol synthase2	EC:2.4.1.123	Pmo06G027030
GSTU19	Glutathione s-transferase tau 19	EC:2.5.1.18	Pmo04G013080
HA1	H(+)-ATPASE 1	EC:7.1.2.1	Pmo03G002540
HPR	Hydroxypryruate reductase	EC:1.1.1.29	Pmo07G023950
NCED3	Nine-cis-exoxycarotenoid dioxygenase 3	EC:1.13.11.51	Pmo04G013330
PHS1	α-Glucan phosphorylase 1	EC:2.4.1.1	Pmo03G022850
SELO	Selenoprotein O	EC:2.7.7.108	Pmo06G001270
SUS3	Sucrose synthase 3	EC:2.4.1.13	Pmo01G010740, Pmo08G022980
UBC33	Ubiquitin-conjugating enzyme 33	EC:2.3.2.23	Pmo03G002630
UBC34	Ubiquitin-conjugating enzyme 34	EC:2.3.2.23	Pmo03G002630
WOL	Wooden leg	EC:2.7.13.3	Pmo01G029440

## 4. Conclusion

Here, we present a high-quality, chromosome-level *P. mongolica* genome assembly encompassing eight pseudochromosomes with a total length of 233.17 Mb. Within this highly complete genomic assembly, we identified 23,798 protein-coding genes, 625 tRNAs, 832 rRNAs, 217 miRNAs, and 283 snRNAs. Phylogenetic analysis based on 1,538 single-copy orthologous genes revealed that *P. mongolica* and *P. persica* are the most closely related, diverging from *P. dulcis* 4.2 Mya. Ks and 4DTv analyses indicated that the *P. mongolica* genome experienced two WGD events. These results suggest that the substantial increase in LTR-RT insertions and tandem gene duplications within the *P. mongolica* genome may have contributed to the expansion of its genome and its adaptation to arid environments. We also identified a number of candidate genes involved in drought resistance and fatty acid biosynthesis. These candidate genes are likely to prove useful for the future studies of drought tolerance and fatty acid biosynthesis in *P. mongolica*, and will provide important genetic resources for molecular breeding and improvement experiments in *Prunus* species. This high-quality reference genome will also accelerate the study of the adaptation of xerophytic plants to drought.

## Supplementary data

Supplemental data are available at *DNARES* online.

dsad012_suppl_Supplementary_Figures

dsad012_suppl_Supplementary_Tables

## Data Availability

The genome assembly and raw sequencing data for *P. mongolica* have been deposited to the National Genomics Data Center (NGDC, https://ngdc.cncb.ac.cn/),^85,86^ with the project number of PRJCA013466 and the genome accession number of GWHBQDV00000000.1. These data were also deposited to NCBI with the project number of PRJNA930130. The detailed information of annotation are available on FigShare at the link: https://doi.org/10.6084/m9.figshare.22214515.v1.
