# Supplementary material for: Chromosome-level genome assembly of an endangered plant Prunus mongolica using PacBio and Hi-C technologies
Source: DNA Res. 2023 May 23;30(4):dsad012. doi: 10.1093/dnares/dsad012 (PMC10254739; doi:10.1093/dnares/dsad012)
Supplement: dsad012_suppl_Supplementary_Figures [file dsad012_suppl_supplementary_figures.pdf]

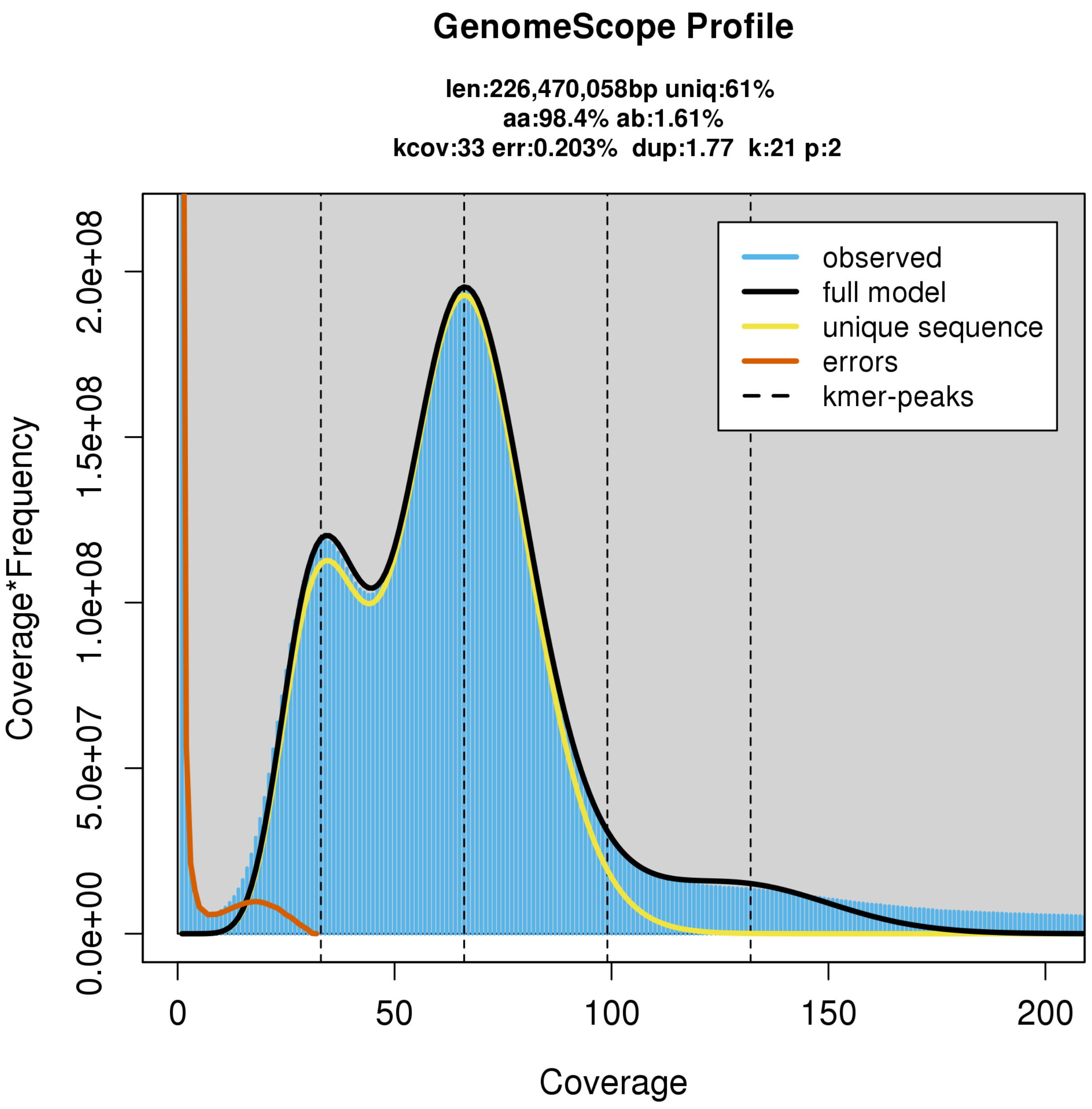

K-mer	*K*-mer number	Peak depth	Genome size (Mb)	Used bases	Used reads	Heterozygous Ratio (%)	repeat sequences (%)
21	1,574,319,910	33	226.47	18,176,486,855	121,176,579	1.61	39.01

Supplemental Figure S1. Genome size and heterozygosity analysis based on k-mer analysis (K=21)

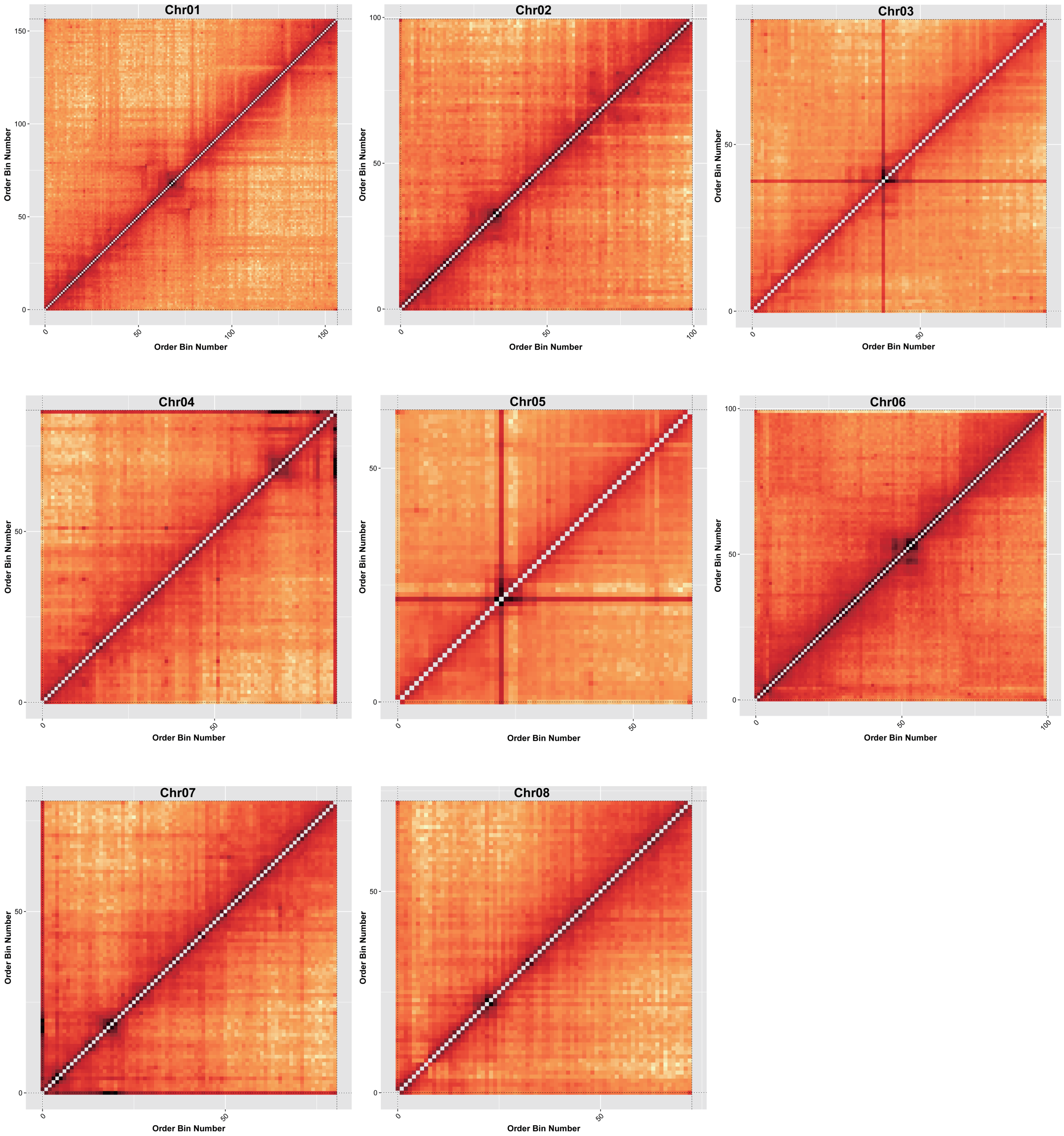

Supplemental Figure S2. Heatmap showing Hi-C interactions of all *P. mongolica* pseudochromosomes at a resolution of 100 kb.

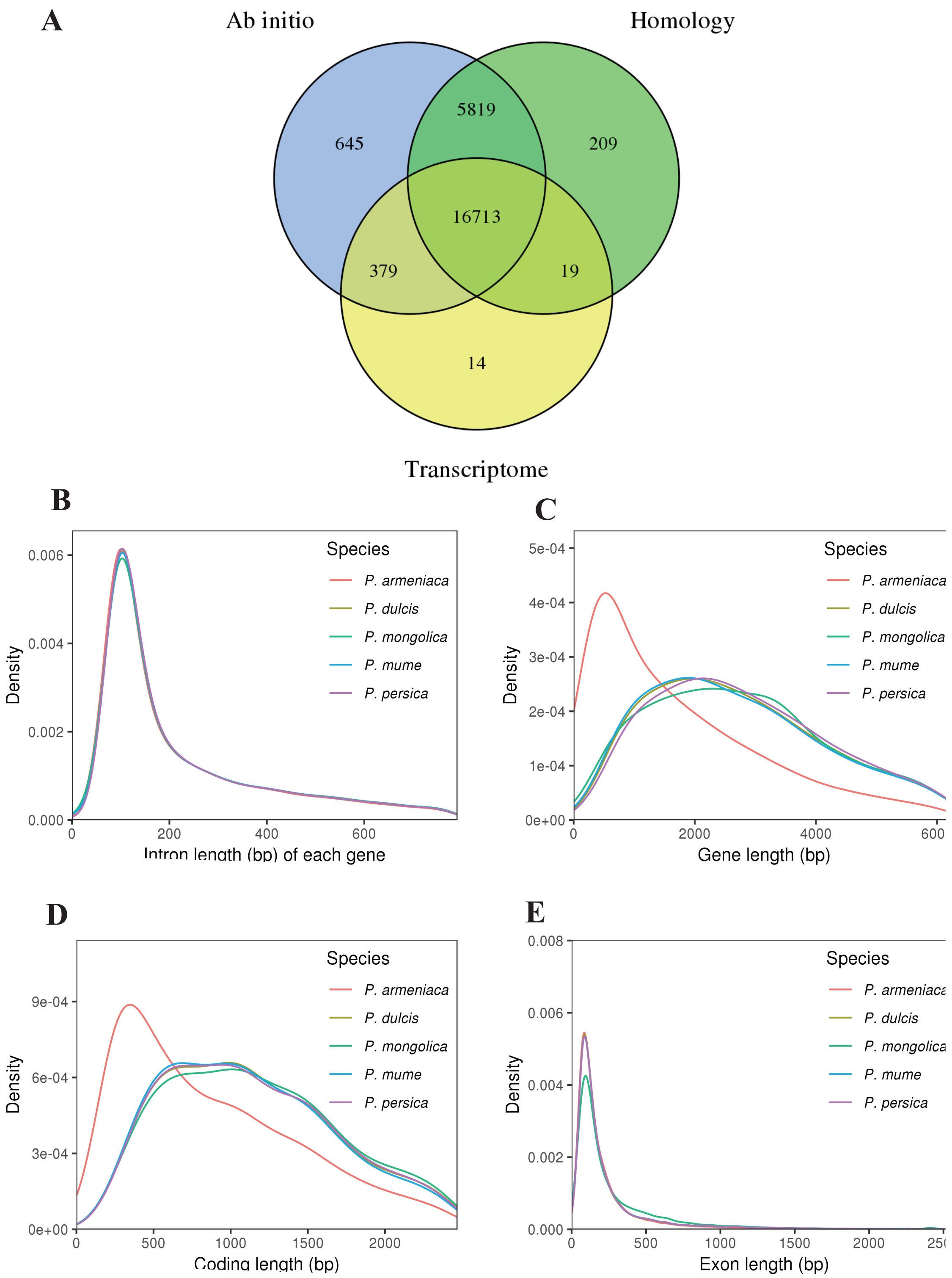

Supplemental Figure S3. **(A)** Venn diagram showing protein-coding genes were predicted on the basis of 3 different strategies. **(B)** The coding length of protein-coding genes in *P. mongolica* and related species. **(C)** The gene length of protein-coding genes in *P. mongolica* and related species. **(D)** The intron length of protein-coding genes in *P. mongolica* and related species. **(E)** The exon length of protein-coding genes in *P. mongolica* and related species.

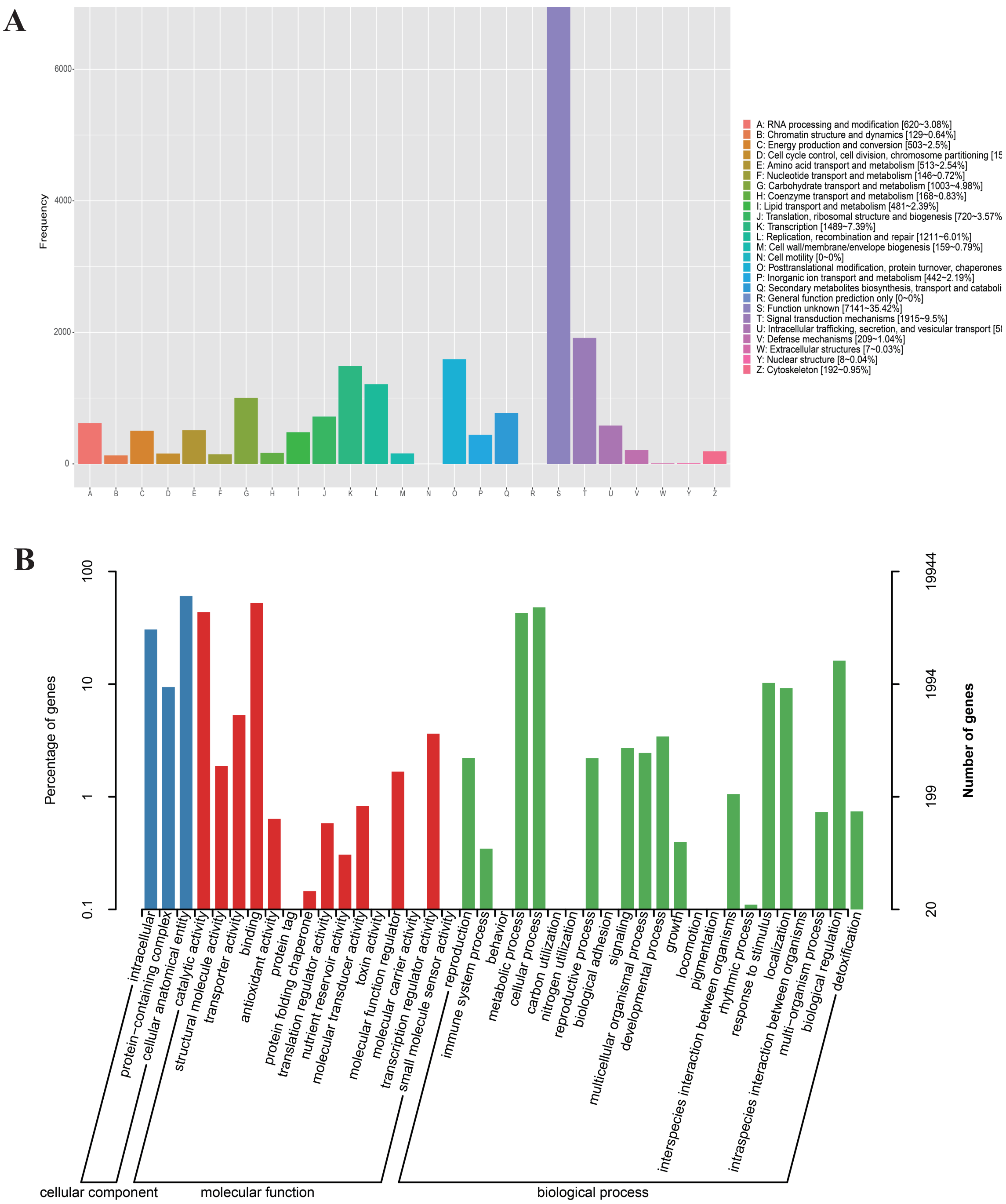

Supplemental Figure S4. (A) eggNOG enrichment analysis of protein-coding genes in *P. mongolica*. **(B)** GO enrichment analysis of protein-coding genes in *P. mongolica*.

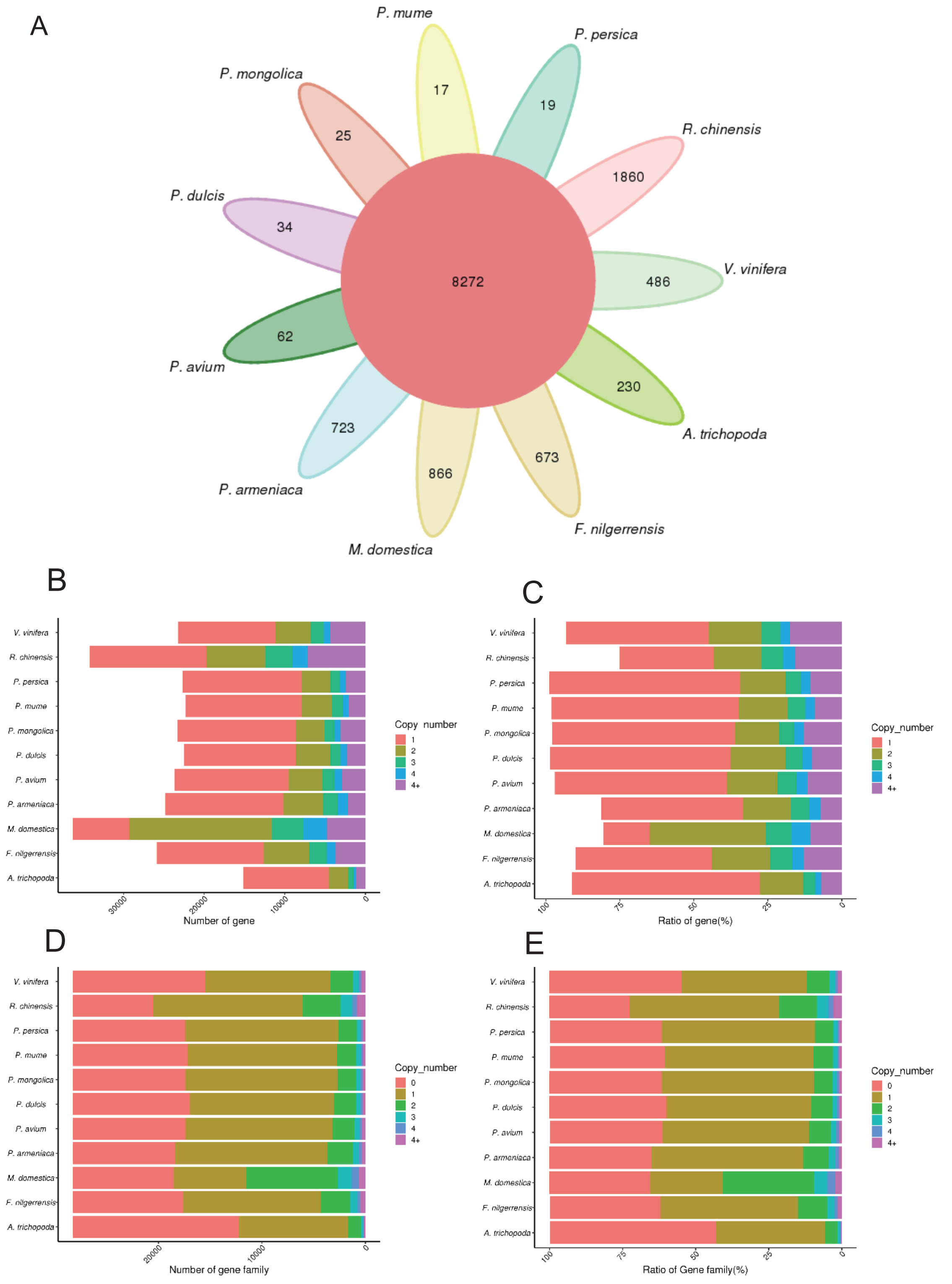

Supplemental Figure S5. Cluster analysis of gene and gene families in *P. mongolica* and other 10 related species.(A) Petal diagram of gene families cluster in 11 species. **(B)** The number of copies distribution of all genes in 11 species. **(C)** The ratio of copies distribution of all genes in 11 species. **(D)** The number of copies distribution of all gene families in 11 species. **(E)** The ratio of copies distribution of all gene families in 11 species.

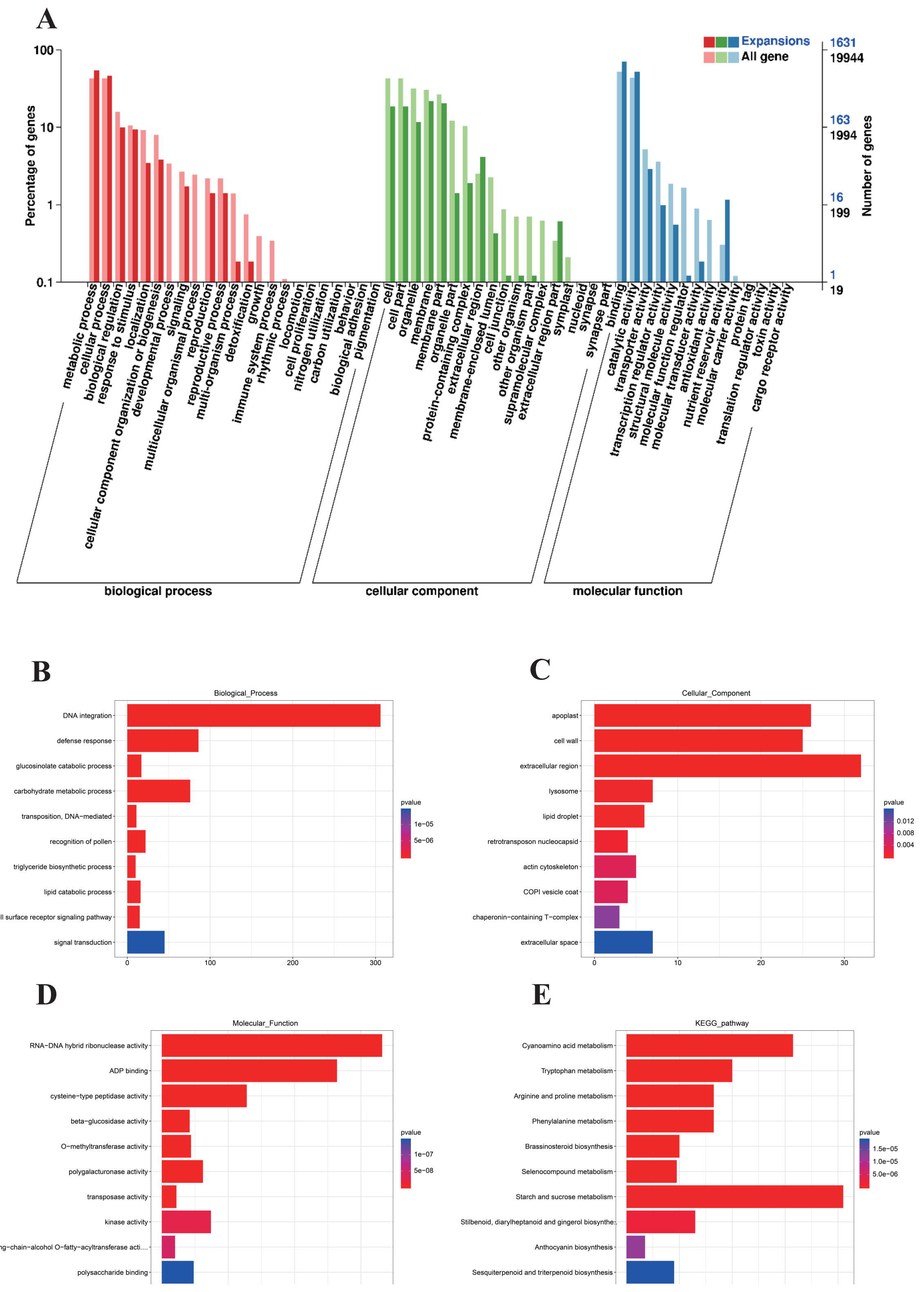

Supplemental Figure S6. Cluster analysis of expansion genes in *P. mongolica*. **(A)** GO enrichment analysis of expansion genes in *P. mongolica*.**(B)** Biological process enrichment analysis of expansion genes in *P. mongolica*. **(C)** Cellular component enrichment analysis of expansion genes in *P. mongolica*. **(D)** Molecular function enrichment analysis of expansion genes in *P. mongolica*. **(E)** KEGG enrichment analysis of expansion genes in *P. mongolica*.

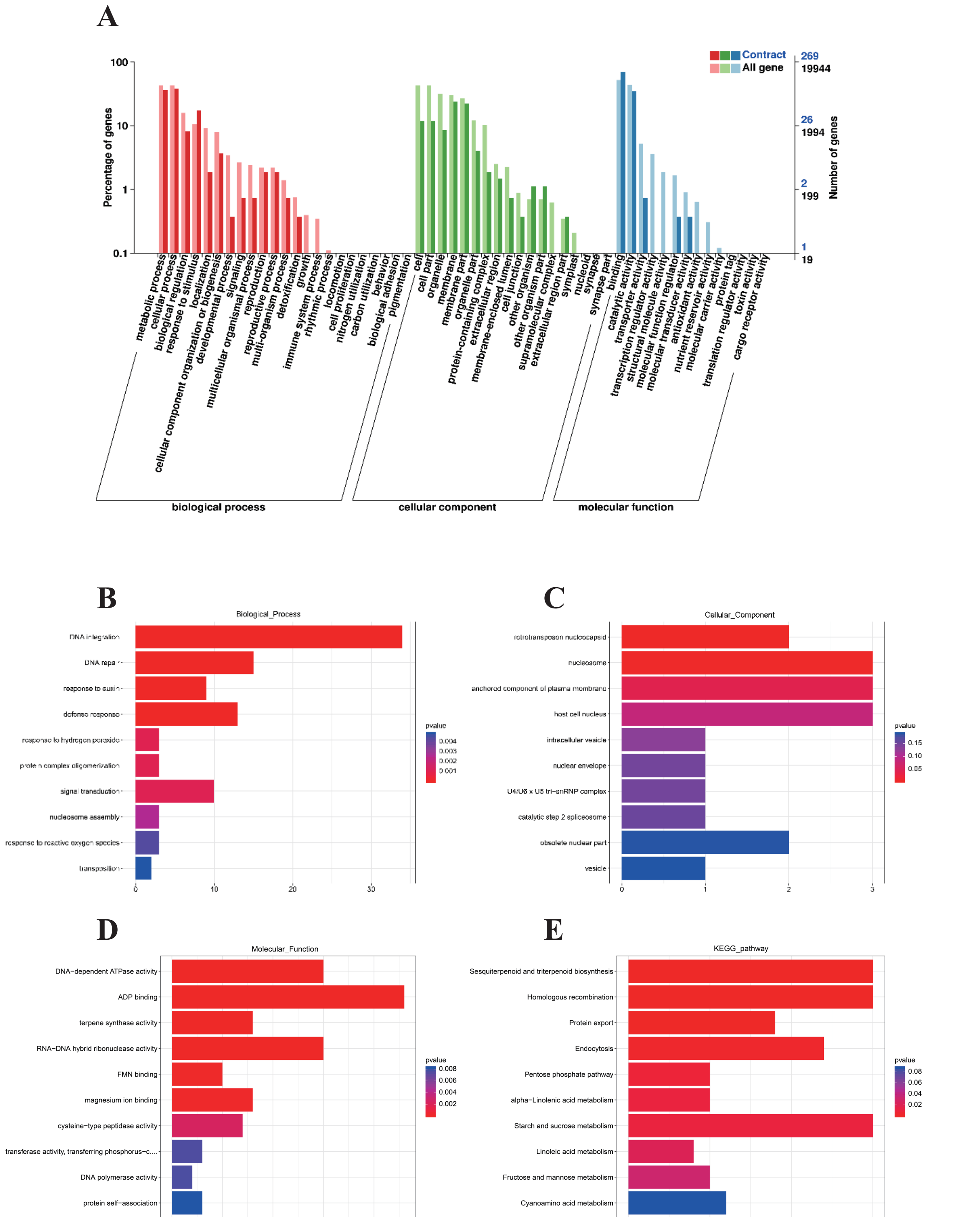

Supplemental Figure S7. Cluster analysis of contraction genes in *P. mongolica*. **(A)** GO enrichment analysis of contraction genes in P. mongolica. **(B)** Biological process enrichment analysis of contraction genes in P. mongolica. **(C)** Cellular component enrichment analysis of contraction genes in P. mongolica. **(D)** Molecular function enrichment analysis of contraction genes in P. mongolica. **(E)** KEGG enrichment analysis of contraction genes in P. mongolica.

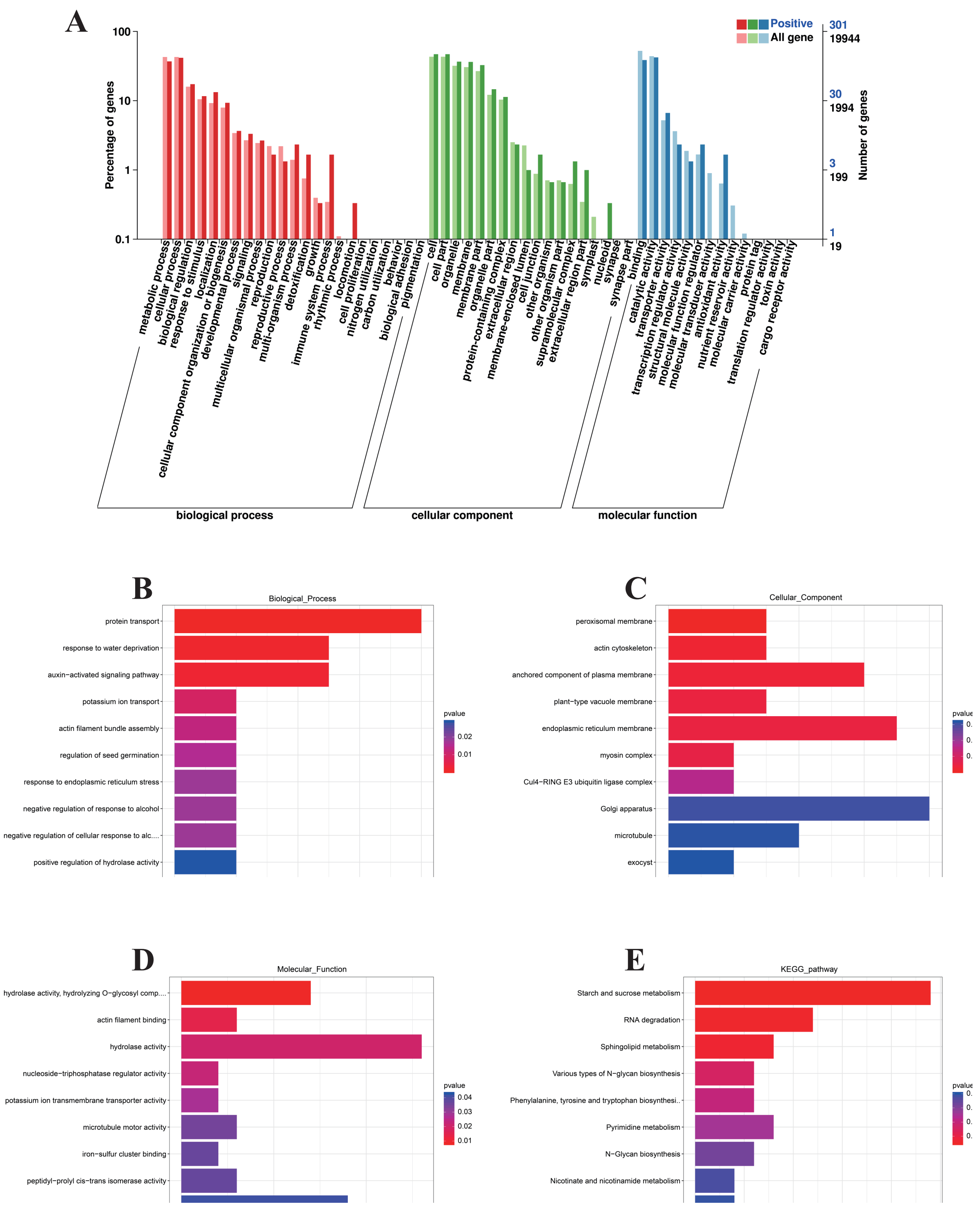

Supplemental Figure S8. Cluster analysis of Positively selected genes in *P. mongolica*. **(A)** GO enrichment analysis of Positively selected genes in *P. mongolica*.**(B)** Biological process enrichment analysis of Positively selected genes in *P. mongolica*. **(C)** Cellular component enrichment analysis of Positively selected genes in *P. mongolica*. **(D)** Molecular function enrichment analysis of Positively selected genes in *P. mongolica*. **(E)** KEGG enrichment analysis of Positively selected genes in *P. mongolica*.

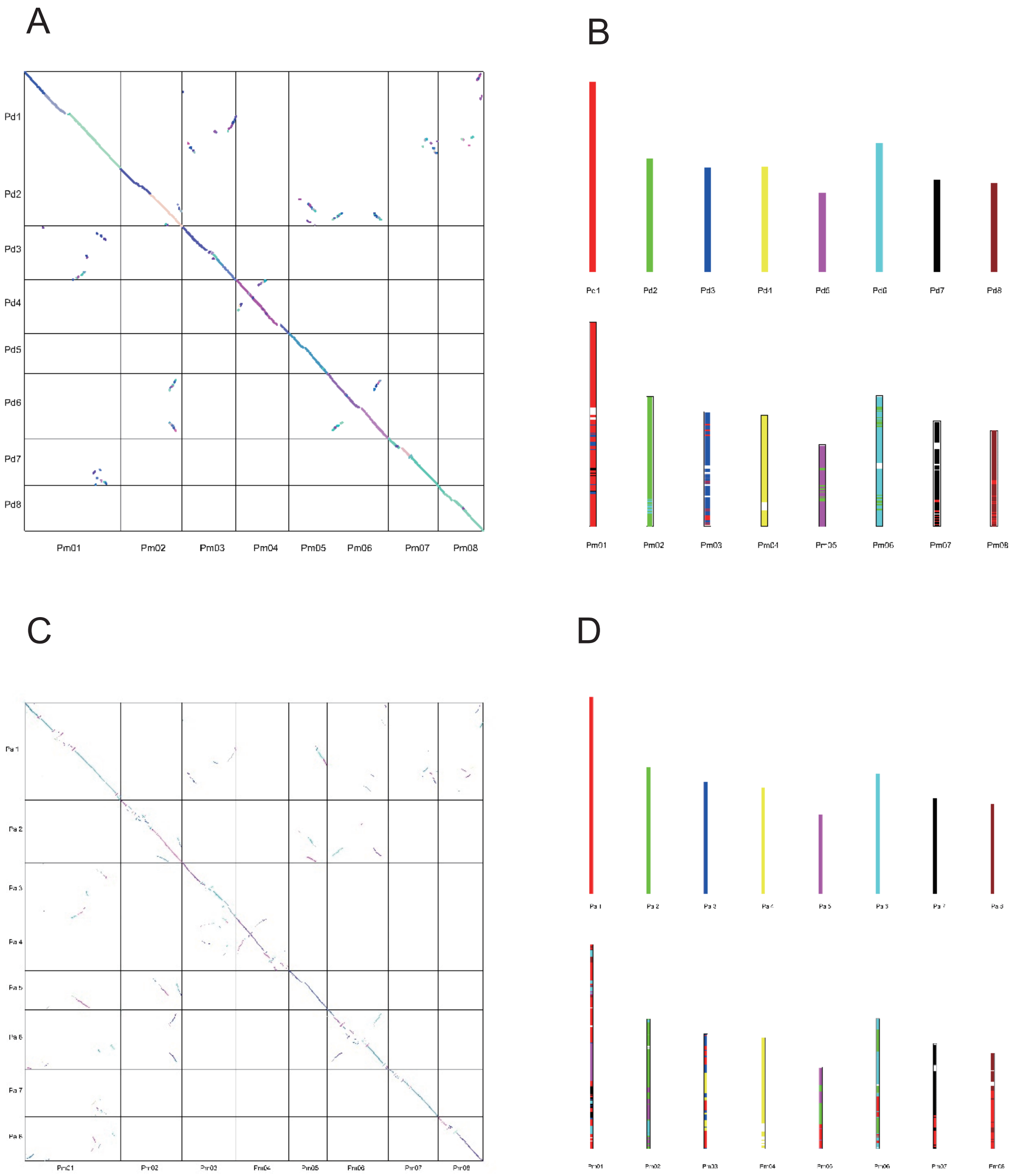

Supplemental Figure S9. Visualization of collinear blocks between *P. mongolica-P. dulcis* and *P. mongolica -P. armeniaca.* (A) The dot graphs of synteny blocks between *P. mongolica* and *P. dulcis*. Dots closest to the diagonal line represent collinearity between the two genomes with < 10 Kb fragments filtered out. **(B)** The bar graphs of synteny blocks between *P. mongolica* and *P. dulcis*. **(C)** The dot graphs of synteny blocks between *P. mongolica* and *P. armeniaca*. Dots closest to the diagonal line represent collinearity between the two genomes with < 10 Kb fragments filtered out. **(D)** The bar graphs of synteny blocks between *P. mongolica* and *P. armeniaca*.
